# Effect of folic acid on bone metabolism: a randomized double blind clinical trial in postmenopausal osteoporotic women

**DOI:** 10.1186/s40199-014-0062-9

**Published:** 2014-09-16

**Authors:** Pooneh Salari, Mohammad Abdollahi, Ramin Heshmat, Hamidreza Aghaei Meybodi, Farideh Razi

**Affiliations:** Medical Ethics and History of Medicine Research Center, Tehran University of Medical Sciences, 23# 16 Azar Ave, Keshavarz Blvd, 1417633114 Tehran, Iran; Faculty of Pharmacy and Pharmaceutical Sciences Research Center, Tehran University of Medical Sciences, Tehran, Iran; Endocrinology and Metabolism Research Institute, Tehran University of Medical Sciences, Tehran, Iran

**Keywords:** Homocysteine, Folic acid, BMD, Bone biomarkers, Osteoporosis, Clinical trial

## Abstract

**Background:**

In spite of several studies, the impact of homocysteine level and folic acid supplementation on bone metabolism is yet to be recognized. In this registered clinical trial (IRCT2014042217385N1), we aimed to find out the power of 6-month folic acid supplementation on homocysteine level and bone metabolism.

**Methods:**

Forty postmenopausal osteoporotic women (50 to 87 years) were enrolled in the study. All participants were randomized to receive folic acid 1 mg (n = 17) or placebo (n = 14). At baseline, 3 months, and finally 6 months post intervention, the level of homocysteine, vitamin B12, and bone biomarkers were measured.

**Results:**

Both groups were similar at baseline. The homocysteine decreased in both groups but statistically non-significant (*P* > 0.05). The changes of the serum level of vitamin B12, osteocalcin, and β cross laps were significant between groups after 6 months (*P* ≤ 0.05).

**Conclusion:**

The trend of changes of bone biomarkers after 6 months folic acid supplementation shows that homocysteine concentration and/or folic acid supplementation have impact on the rate of bone metabolism. However, further investigations by larger sample size and differentiating age and gender are still needed to clarify the exact role of folate, homocysteine and vitamin B12.

## Introduction

Osteoporosis, the senile chronic disease is mainly caused by estrogen deficiency, while, environmental and lifestyle factors as well as metabolic and genetic disorders are contributing. As a chronic senile inflammatory disease, it is believed that osteoporosis has common pathophysiological basis with cardiovascular diseases [1]. Hyperhomocysteinemia is considered as an independent risk factor for several chronic senile diseases and conditions including cardiovascular diseases, and Alzheimer disease [2,3]. As the number of devastating consequences in osteoporotic patients is increasing worldwide, determining the possible risk factors and their elimination is crucial. Keeping this in mind, recent studies have shown the possible role of platelets, inflammation, and homocysteine as well as the role of specific medications in the common pathophysiologic pathway of cardiovascular diseases and osteoporosis [4-10]. Accordingly, the role of hyperhomocysteinemia in the pathogenesis of osteoporosis has been considered as a focal point. Serum concentration of homocysteine is inversely related to those of vitamin B12 and folic acid [11]. From one point during menopause the serum level of homocysteine is increased and from the other point hyperhomocysteinemia is linked with risk of fracture. Folate supplementation may modify the serum concentration of homocysteine, and of greater importance may affect fracture risk, because high homocysteine levels increase the risk of fracture [12]. To our knowledge, there is no enough data about the effect of folate supplementation and homocysteine lowering therapy on bone metabolism and fracture risk. Our previous systematic review showed the possible impact of high homocysteine level on bone quality via induction of osteoclast related bone resorption [3]. With all these in mind, the exact role of homocysteine level and folic acid supplementation in regulation of bone metabolism is yet to be elucidated. Based on the implication of bone biomarkers in determination of bone metabolism in osteoporotic patients in a short period of time we aimed to examine the effect of folic acid supplementation on serum level of homocysteine and bone metabolism in postmenopausal osteoporotic women in a randomized, double-blind, placebo-controlled study. For this purpose, we selected key indicators such as bone specific alkaline phosphatase (BALP) from enzymes, osteocalcin (OC) from bone protein markers, β cross laps (CTX) and pyridinoline (PYD) from collagenous bone resorption markers to evaluate bone metabolism. All selected bone biomarkers are easily changed within 3–6 months treatment and usually used for monitoring effectiveness of treatment. Furthermore measuring OC and CTX both together is considered as a reasonable choice for evaluating bone degradation and synthesis [13].

## Methods

### Subjects

Forty postmenopausal osteoporotic women who referred to the Diabetes and Metabolic Disorders Clinic were enrolled in the double blind randomized clinical trial. Osteoporosis was diagnosed by the mean of dual energy X-ray absorptimetry (Hologic®) at femur neck and lumbar vertebrae in all participants. Osteoporosis was defined on the basis of WHO criteria as having BMD ≥ 2.5 SD below the normal mean for young adult women or T-sore ≤ −2.5. Participants profile including age, age at menopause, habit of smoking, alcohol consumption, past medical history and use of medications were collected. Participants with a history of cancer, acute infection, endocrinology diseases, taking medications affecting bone metabolism, such as corticosteroids, gonadotropin releasing hormone (GnRH) analogues, anticonvulsant drugs, heparin, aluminum containing antacids, thyroid hormones and anti-osteoporosis medications were excluded (Table 1). The study was registered in the Iranian Registry of Clinical Trials with the code of IRCT2014042217385N1.

**Table 1:** **Exclusion criteria**

**Exclusion criteria**	
Cancer	Medications:
Acute infection	Corticosteroids
Endocrinologic diseases	Gonadotropin releasing hormone (GnRH) and its analogues Antiosteoporotic medications (bisphosphonates, calcium and vitamin D, teriparatide, calcitonin, etc)
Taking medications affecting bone metabolism	Anticonvulsants
	Heparin
	Aluminum containing antacids
	Thyroid hormones
	Selective serotonin reuptake inhibitors (SSRIs)

The study was approved by the Ethics Committee of the Endocrinology and Metabolism Research Institute of Tehran University of Medical Sciences. All participants signed the written informed consent after receiving complete information about the study and their contribution.

### Experimental design

**Figure 1:**
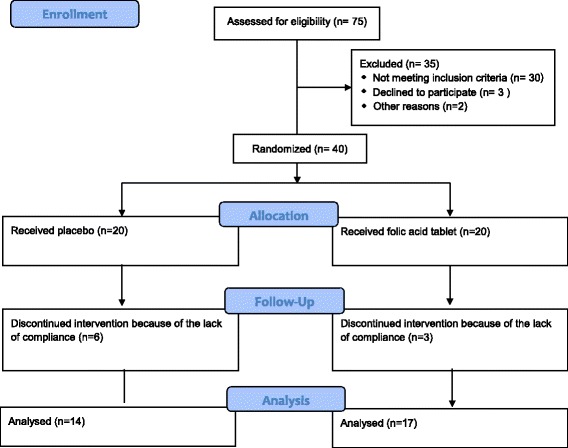
**Consort flowchart.**

All participants were randomly assigned to folic acid or placebo. The patients’ assignments were double blind. The method of randomization was simple. Because of few dose–response studies, the most proper dose of folic acid to lower homocysteine concentration is not fully clarified. In a dose–response trial, no significant relation was observed in reduction of homocysteine level when various doses of folic acid were used [8]. Also, the Homocysteine Lowering Trialists Collaboration meta-analysis showed no significant dose-dependent response in the dose range (800–2000 μg/day) of folic acid [14]. Noteworthy, pharmacological doses of folic acid (5 mg/day) may increase the risk of adverse effects [15]. Therefore, present study was designed to administer folic acid at dose of 1 mg/day as a supplement (Figure 1).

Both folic acid and placebo tablets were completely similar in taste, texture and appearance and were produced by Rooz Daru Company (Iran). All participants were asked to inform the clinic in the case of any change in their lifestyle, medication or occurrence of any side effect. Over the study period, in each visit, the patients were asked to feel free to exit from the study if they did not comply with the described protocol.

### Laboratory studies

Blood and urine samples were taken at baseline and after 3 and 6 months. All samples were collected in the early morning and fasting condition. Blood samples were centrifuged at 10000 × g for 10 min and kept in the fridge at −70°C until analysis. Serum levels of bone biomarkers including OC, BALP, homocysteine, vitamin B12 and urinary concentrations of PYD and β cross laps were measured. Serum OC and β cross laps were quantified by N-MID®Osteocalcin ELISA and Urine BETA CrossLaps® ELISA Immuno Diagnostic Systems (IDS) (Germany), respectively. Urinary PYD and serum level of BALP were measured by MICROVUE PYD EIA® and MICROVUE BAP EIA® Kits from QUIDEL Corporation (Germany), respectively. Serum level of homocysteine was quantified with enzymatic assay by Axis Shield® and serum level of vitamin B12 was measured by the method of chemiluminescence By Diasorin Liaison®. Intra- and inter-assay coefficients of variation (CV) were 2.2% and 5.1% for osteocalcin, 3.9% and 6.9% for β cross laps, 11.2% and 9.9% for PYD, 7.6% and 5.8% for BALP, and 4.4% and 2.2% for homocysteine.

### Statistical analysis

The StatsDirect version 3.0.117 was used for statistical analysis. Data are expressed as mean ± SD. Normal distribution of data was confirmed by Kolmogorov-Smirnov test. Baseline values and values after supplementation were compared using T-test. Comparison between groups before and after supplementation was performed using General Linear Model (repeated measure) test. The Pearson correlation test was used for correlations. *P* ≤ 0.05 was considered significant.

## Results

The study performed on 40 postmenopausal women, of those 31 participants completed the study (response rate = 77.5%). Three participants from the treatment group and 6 participants from the control group withdrew the study because of lack of compliance (Figure 1). Table 2 shows the demographic data of all participants. No lifestyle or medication change as well as side effect was recorded. The distribution of data was normal. No statistically significant difference was found between groups at baseline (Table 2). The average baseline homocysteine and vitamin B12 levels were 12.77 μmol/L and 605.72 pg/ml, respectively. The average baseline level of BALP, OC and CTX was 30.5 U/L, 15.48 ng/ml, and 2.74 (μg/mmol Cr), respectively which were in the normal range. The average baseline urinary PYD level of 43.27 (μg/mmol Cr) was higher than normal level.

**Table 2:** **Demographic data and baseline characteristics of study participants**

	**Normal values**	**Treatment group (17)**	**Control group (14)**	***P*** **value**
Age (yr)		63.8 ± 8.1	64.2 ± 7.3	0.870
BMI (kg/m^2^)		25.2 ± 4.5	26.7 ± 3.1	0.290
Age at menopause (yr)		48.8 ± 4.6	46.6 ± 4.7	0.196
Lumbar BMD (g/cm^2^)		0.67 ± 0.16	0.71 ± 0.06	0.370
Femoral BMD		0.59 ± 0.07	0.60 ± 0.06	0.709
Lumbar T-score		−3.11 ± 0.43	−3.01 ± 0.62	0.615
Femoral T-score		−2.32 ± 0.63	−2.29 ± 0.46	0.832
Homocysteine (μmol/L)	5-20	11.70 ± 6.63	14.07 ± 3.60	0.242
Vit B12 (Pg/ml)	160-970	745.70 ± 623.07	435.75 ± 245.25	0.091
OC (ng/ml)	12.8-55.0	15.34 ± 6.94	15.66 ± 9.99	0.916
BALP (U/L)	14.2-42.7	29.94 ± 11.02	31.17 ± 11.90	0.767
UPYD (μg/mmol cr)	16.0-37.0	42.99 ± 11.67	43.61 ± 11.14	0.882
U β cross laps (μg/mmol cr)	0.73-7.07	2.45 ± 1.58	3.10 ± 1.75	0.285

All data are presented as Mean ± SD. Yr = year; BMI = body mass index; BMD = bone mineral density; Vit B12 = vitamin B12; OC = osteocalcin; BALP = bone alkaline phosphatase; UPYD = urine pyridinium cross links; U β cross laps = urine β cross laps.

Table 3 presents the biochemical results after supplementation. The serum level of homocysteine decreased in both groups along the study. No significant change was found in each group (*P* = 0.22 in treatment group versus *P* = 0.26 in the control group) and between groups at 6 months (*P* = 0.06).

**Table 3:** **Statistics for serum concentrations of bone biomarkers in both groups in the study period**

	**Treatment group (N = 17)**			***P*** **value**	**Control group (N = 14)**			***P*** **value**
	**Baseline**	**3 months**	**6 months**		**Baseline**	**3 months**	**6 months**	
Homocysteine (μmol/L)	11.70 ± 6.63	11.52 ± 4.47	10.17 ± 4.09	0.226	14.07 ± 3.60	14.50 ± 4.78	13.21 ± 4.54	0.266
CI 95%	8.55-14.85	9.40-13.65	8.23-12.12		12.18-15.96	11.99-17.00	10.83-15.59	
Vit B12 (Pg/ml)	745.70 ± 623.07	517.17 ± 260.77	655.17 ± 460.18	0.189	435.75 ± 245.25	375.50 ± 475.02	235.42 ± 57.96	0.173
CI 95%	449.51-1041.89	393.21-641.14	436.42-873.94		307.69-564.23	126.66-624.33	205.70-265.79	
OC (ng/ml)	15.34 ± 6.94	14.10 ± 6.13	13.84 ± 4.87	0.384	15.66 ± 9.99	16.71 ± 9.92	17.70 ± 10.03	0.460
95% CI	12.03-18.64	11.18-17.02	11.53-16.16		10.43-20.90	11.51-21.91	12.44-22.96	
BALP (U/L)	29.94 ± 11.02	30.82 ± 9.66	31.74 ± 9.30	0.614	31.17 ± 11.90	32.14 ± 14.35	34.46 ± 15.10	0.326
95% CI	24.70-35.19	26.23-35.42	27.33-36.17		24.94-37.41	24.62-39.66	26.55-42.37	
UPYD (μg/mmol cr)	42.99 ± 11.67	47.18 ± 9.99	46.61 ± 17.60	0.369	43.61 ± 11.14	45.11 ± 11.04	51.26 ± 15.56	0.107
95% CI	37.44-48.54	42.43-51.93	38.25-54.99		37.78-49.45	39.32-50.90	43.11-59.41	
U β cross laps (μg/mmol cr)	2.45 ± 1.58	2.43 ± 1.22	2.77 ± 1.86	0.358	3.10 ± 1.75	3.25 ± 1.92	3.68 ± 2.40	0.398
95% CI	1.70-3.20	1.85-3.01	1.90-3.66		2.18-4.03	2.24-4.27	2.42-4.94	

All data are presented as Mean ± SD. CI = confidence interval; Vit B12 = vitamin B12; OC = osteocalcin; BALP = bone alkaline phosphatase; UPYD = urine pyridinium cross links; U β cross laps = urine β cross laps.

In the treatment group, the serum level of vitamin B12 decreased in the first three months followed by an increase after 6 months, while in the control group it decreased during the study period. No significant change in the serum level of vitamin B12 was found in each group (*P* = 0.189 and *P* = 0.173). After 6 months serum concentration of vitamin B12 was significantly lower in the control group compared with the treatment group (*P* = 0.01) (Table 3).

The serum level of OC showed a non-significant increase and a decrease in the control group (*P* = 0.460) and treatment groups, respectively (*P* = 0.384). After 6 months, serum level of OC was significantly higher in the control group compared with the treatment group (*P* = 0.034; Table 3).

The serum level of BALP showed a non-significant increase in the treatment (*P* = 0.614) and control (*P* = 0.326) groups. No significant difference was observed between groups after 6 months (*P* = 0.543; Table 3).

The urinary levels of PYD and β cross laps non-significantly increased in both groups, more prominently in the control group (Table 3). After 6 months, the increase in the level of β cross laps in the control group was higher than that of treatment group (*P* = 0.01).

Baseline level of PYD and BALP in the control group was inversely correlated with BMD and T-score of the lumbar spine (r = −0.53, *P* = 0.05) and (r = −0.59, *P* = 0.02), respectively. In the control group, the level of β cross laps at baseline and after 6 months showed a negative correlation with BMD and T-score of the lumbar spine (r = −0.61, *P* = 0.02) and (r = −0.61, *P* = 0.01), respectively. In addition homocysteine concentration at the end of the study was inversely correlated with BMD and T-score of femur (r = −0.65, *P* = 0.01) and (r = −61, *P* = 0.01), respectively.

## Discussion

In this study we aimed to evaluate the effect of folate supplementation on homocysteine level and bone metabolism in postmenopausal osteoporotic women. In addition we presumed correlations between homocysteine, bone biomarkers and bone specific characteristics including BMD and T score. For this purpose we measured each participant’s BMD and T-score of the femur and lumbar spine as well as bone biomarkers. In fact measuring femoral BMD has been considered as the standard of choice for osteoporosis because this site shows the fracture risk similar or higher than that of other sites [16].

Our results showed that supplementation with 1 mg folic acid non-significantly decreases homocysteine level; however reduction of homocysteine in the treatment group was more than control group. According to the Table 1, the mean homocyteine level of all participants at baseline was higher than 10 μmol/L which is a mild elevation [17]. There are several studies which indicate significant homocysteine lowering effect of folic acid in doses 400–800 μg per day [18-20]. It is noteworthy to consider that participants of those studies were supplemented with different doses of vitamin B12 (9–1000 μg/day) in addition to folic acid. We suppose that lack of significant effects of folic acid supplementation on homocysteine level is because of mildly elevated values of homocysteine and normal levels of vitamin B12 in both groups at the baseline. Our thought is in agreement with that of Homocysteine Lowering Trialists Collaboration [14]. In addition to serum homocysteine level, folate status may be affected. There is a report that in healthy older adults with normal serum folate level, folic acid supplementation cannot reduce homocysteine level while in those with low serum folate supplementation it may lower homocysteine concentration [21]. It should be noted that in our study and most of other studies, blood samples were taken in the fasting state while a study indicated that about 50% of participants show hyperhomocysteinemia after methionine load [22]. Therefore it is suggested that fasting blood sample lacks enough sensitivity for measuring serum homocysteine. Likewise racial differences which affect polymorphism of the methylenetetrahydrofolate reductase (MTFHR) gene may affect homocysteine metabolism and should be taken into account in future studies.

In the control group the hemocysteine level at the end of the study was negatively correlated with BMD and T score of femur at baseline (r = −0.65, *P* = 0.01). Although several studies indicated significant higher levels of homocysteine in osteoporotic women and its inverse relationship with BMD of total femur [23-25] or the risk of fracture [26,27], other investigations did not confirm the association between homocysteine and BMD and/or bone markers [28,29]. This is because of a weak correlation between homocysteine and BMD that failed to be detected in small sample size. A dose–response relationship between homocysteine concentration and bone loss in men and premenopausal women has been reported [30]. In a case–control study in the Women’s Health Initiative Observational Study, the modest association of high homocysteine levels with increased risk of hip fracture was found [12]. On the other hand, a high level of homocysteine or low level of vitamin B12 and folate were not found as the risk factors of vertebral fractures in postmenopausal women. However, the relationship between levels of homocysteine and vitamin B12 and low BMD was recognized [31]. In a cohort study, association of high homocysteine concentration and higher bone loss over 4 years in postmenopausal women was found [32].

Our data showed significant difference in the serum level of vitamin B12 after 6 months and its serum level was significantly lower in the control group at the end of the study (*P* = 0.01).

Vitamin B12 acts as a cofactor for transformation of homocysteine to methionine. So both folic acid and vitamin B12 may reduce serum homocysteine. Vitamin B12 seems to be involved in osteoblast activity and formation of bone [33], and inhibits osteoclast function [34]; however, the exact mechanism of vitamin B12 in influencing bone density is not fully understood.

According to the Framingham Osteoporosis Study, serum level of vitamin B12 less than 250 pg/ml is considered as vitamin B12 deficiency and is associated with lower BMD [35]. The participants in our study had normal serum level of vitamin B12 and after 6 months its mean interestingly reached 233.45 pg/ml in the control group.

We found no correlation between vitamin B12 and bone markers or BMD which is in agreement with other study which showed no relationship between vitamin B12 supplementation and level of BALP in Mexican women [36].

It was found that vitamin B12 and folate are correlated with BMD in postmenopausal women and the possibility of involvement of a mechanistic link was suggested [37].

We observed non-significant increase and decrease in the level of OC in the control and treatment groups, respectively; however we found a significant difference between groups. To date no study has evaluated the pattern of alteration of OC in relation to homocysteine.

The level of BALP over the study period showed non-significant increase in both groups, more profoundly in the control group.

Homocysteine interacts with lysyl oxidase in collagen post-translational modifications and cross linking. Therefore it influences formation of the cross-link [38]. In addition to bone formation markers, bone resorption markers including PYD and β cross laps were assessed in the present study. Our results showed parallel non-significant increase in the levels of both PYD and β cross laps in both groups. Furthermore the level of β cross laps was significantly higher in the control group. According to the higher level of all measured bone biomarkers in the control group after 6 months, it is suggested that folic acid supplementation reduces serum level of homocysteine which follows by suppression of bone metabolism. However because of small sample size, the power of our study does not permit to prove our hypothesis. Not only our study but also studies with longer duration up to 2 years could not show a significant effect of folic acid supplementation on bone markers [34,39]. Despite a significant reduction of homocysteine levels with folic acid and vitamin B12 supplementation, no significant impact of vitamin B supplementation on bone markers was found in another study while the final level of CTX and alkaline phosphatase was higher in the control group [17]. In contrast, a study reported a strong correlation between homocysteine and serum c-terminal telopeptide (CTx) levels [40]. The results of a recent longitudinal study showed a positive correlation between homocysteine and total alkaline phosphatase. It was concluded that increase in BALP after hyperhomocysteinemia is the result of induction of bone regeneration after bone resorption [29]. Those results may support our results from this point of view.

Regarding the small sample size and enrolment of patients from one referral center limits extrapolation of the results of the study. Although we asked the participants to follow their routine lifestyle in the study period, our information about their lifestyle is lacking. Noteworthy to consider, all of the participants had mildly elevated serum level of homocysteine which may affect their response to supplementation; also none of the participants were taking calcium and/or vitamin D or other type of supplement.

Our study presents that level of all of the biochemical bone markers showed the highest increase in the control group versus the treatment group in the study period and the changes in OC, and β cross laps were significant between groups. Our hypothesis is that serum level of homocysteine, folate supplementation and serum level of vitamin B12 may have a preventive effect on bone metabolism and inversely affect bone metabolism and its rate, however because of the limited sample size, the potency of our study is not enough to be extrapolated. Therefore further investigations about the impact of folic acid supplementation on the rate of bone metabolism in men and women from different ages with larger sample size is highly recommended.
